# Nucleic Acid Sensors and Type I Interferon Production in Systemic Lupus Erythematosus

**DOI:** 10.3389/fimmu.2013.00319

**Published:** 2013-10-07

**Authors:** Meena Shrivastav, Timothy B. Niewold

**Affiliations:** ^1^Department of Rheumatology, Mayo Clinic, Rochester, MN, USA

**Keywords:** systemic lupus erythematosus, nucleic acid sensor, type 1 interferon, TLR, DNA, RNA

## Abstract

The characteristic serologic feature of systemic lupus erythematosus (SLE) is autoantibodies against one’s own nucleic acid or nucleic acid-binding proteins – DNA and RNA-binding nuclear proteins. Circulating autoantibodies can deposit in the tissue, causing inflammation and production of cytokines such as type 1 interferon (IFN). Investigations in human patients and animal models have implicated environmental as well as genetic factors in the biology of the SLE autoimmune response. Viral/Bacterial nucleic acid is a potent stimulant of innate immunity by both toll-like receptor (TLR) and non-TLR signaling cascades. Additionally, foreign DNA may act as an immunogen to drive an antigen-specific antibody response. Self nucleic acid is normally restricted to the nucleus or the mitochondria, away from the DNA/RNA sensors, and mechanisms exist to differentiate between foreign and self nucleic acid. In normal immunity, a diverse range of DNA and RNA sensors in different cell types form a dynamic and integrated molecular network to prevent viral infection. In SLE, pathologic activation of these sensors occurs via immune complexes consisting of autoantibodies bound to DNA or to nucleic acid-protein complexes. In this review, we will discuss recent studies outlining how mismanaged nucleic acid sensing networks promote autoimmunity and result in the over-production of type I IFN. This information is critical for improving therapeutic strategies for SLE disease.

## Introduction

The normal immune system strikes a delicate balance between defense against foreign invasion and the prevention of misdirected responses against self-antigens. Sometimes, this intricate balance becomes faulty due to genetic, environmental, or other factors leading to breakdown of self-tolerance and the onset of an autoimmune disorder. Systemic Lupus Erythematosus (SLE) is a prototype autoimmune disease that affects the skin, kidney, musculoskeletal, and hematologic systems and is characterized by presence of various autoantibodies against self-components, especially double-stranded DNA (dsDNA) and RNA-binding nuclear proteins. Amongst SLE patients, the female to male ratio is 9:1, suggesting that sex-related factors are important in the development of the disease (1, 2). Many genetic factors have been strongly associated with disease susceptibility (3, 4). Exposure to several viruses and bacterial infections, and also UV light are known to trigger SLE (5). Thus, it is considered that SLE occurs when an environmental trigger acts on a genetically predisposed individual, leading to a loss of tolerance toward native proteins (6). Multiple immune system abnormalities contribute to the pathogenesis of SLE, including abnormal clearance of apoptotic cells and immune complexes, over-production of type I interferon (IFN), reduced thresholds for B and T lymphocyte activation, and production of autoantibodies against self-antigens (7). These autoantibodies are directed against nucleic acids and RNA-binding proteins such as Ro, La, and Sm (8). Tissue damage is mediated in part by deposition of immune complexes in the affected organs, followed by activation of downstream inflammatory pathways mediated by complement and FcR engagement of innate immune cells (9). Viruses such as Cytomegalovirus (CMV), Epstein–Barr (EBV), and Parvovirus B19 are frequently involved as environmental triggers in lupus. Hypomethylated bacterial and viral DNA are potent inducers of immune responses through TLR signaling cascade finally leading to type 1 IFN over-expression, B cell activation, production of autoantibodies, and interleukin (IL)-6 (10).

Many patients with SLE have high circulating levels of type I IFN (11). Some individuals treated with IFN-α for chronic viral infections developed *de novo* SLE that was resolved when IFN-α was withdrawn (12, 13). Additionally, within SLE families abnormally high IFN-α levels have been found clustered (14). A recent genome-wide association study has identified additional novel genetic loci associated with high serum IFN-α in SLE patients (15, 16). Taken together, these data support the idea that genetically determined endogenous elevations in IFN-α predispose to human SLE.

## How Does Lupus Start?

**Figure 1:**
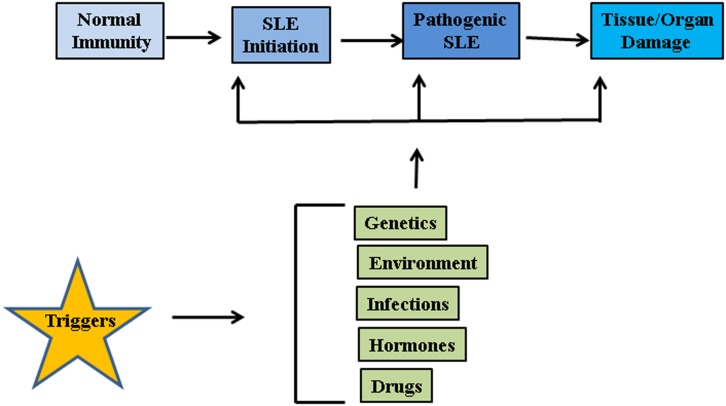
**Factors associated with SLE pathogenesis**. Genetic factors, environmental influences such as radiations, repeated infections, hormonal imbalances, and certain drugs may act on innate immune system and disrupt the intricate balance between protection against foreign invasion and self-defense.

The etiology of lupus is considered to be multifactorial involving multiple genes and environmental factors such as infections, hormones, and drugs (Figure 1) (17). It is considered that unrestrained immune response to apoptotic cells and decreased disposal of apoptotic material are important initiators of the autoimmune response in SLE. Genomic DNA is not accessible to the immune system under standard conditions as it is safely sequestered in the nucleus or in mitochondria under the tight control of DNA damage and repair response systems. However, when cells die through apoptosis, apoptotic bodies containing fragmented cellular material and abnormal surface antigens, circulate in the body enabling the immune system to access new epitopes (18). Under normal conditions cellular mechanisms exist to ensure that apoptotic debris is not immunogenic to self, but these mechanisms can fail. It seems likely that defective clearance of apoptotic material and modifications to DNA such as hypomethylation can promote SLE (19). Recent reports suggest that neutrophil extracellular traps (NETs) are a potent stimulus for type 1 IFN release by plasmacytoid dendritic cells (DCs), and play an important role in propagation of the lupus phenotype (20–23). Neutrophils are specialized immune cells that are rapidly recruited to sites of inflammation in response to microbial infections. One of the mechanisms of neutrophil action is the formation of “NETs” (24). NETs are made of processed chromatin bound to granular and selected cytoplasmic proteins. NETs are released by neutrophils to control microbial infections (24). This release of chromatin is the result of a unique form of cell death, called “NETosis.” Material derived from NETosis can contribute to SLE by serving as source of autoantigen, propagating inflammation, and tissue damage (21, 23, 25, 26). In an interesting recent study, Sangaletti et al. suggested that NETs may provide antigens to DCs and in this way promote immune responses against neutrophil antigens in the autoimmune disease small vessel vasculitis, which is characterized by antibodies against cytoplasmic proteins in neutrophils (23). It is possible that NETs may provide nuclear antigens to immune cells in a similar way in SLE.

Pathways through which our own nuclear material is able to induce pro-inflammatory responses are a topic of active research. At least three distinct types of nucleic acid recognition receptors are recognized: (1) the toll-like receptors (TLRs), which recognize nucleic acids on the plasma membranes and endosomes; (2) the nucleotide binding and oligomerization domain (NOD) receptors (NLRs), which monitor the cytosolic compartment and also interact with TLR pathways; and (3) the retinoid acid inducible gene (RIG)-I-like receptors that recognize RNA or DNA in the cytoplasm (RLRs). Many of these receptors may directly or indirectly participate in the pathogenesis of SLE (27).

## Toll-Like Receptor Mediated Signaling in Lupus

Toll-like receptors are major components of the innate immune system that activate multiple inflammatory pathways and coordinate systemic defense against microbial pathogens. Data from animal models and human patients suggest that improper engagement of TLR pathways by endogenous or exogenous ligands may lead to the initiation of autoimmune responses and tissue injury (28). Endosomal TLRs (TLR-3, -7, -8, and -9) are potent activators of DCs and B cells. TLR-3 is specific for double-stranded RNA (dsRNA), TLRs-7 and -8 for single-stranded RNA (ssRNA), and TLR-9 is specific for dsDNA (29, 30). TLRs are expressed predominantly in DCs, B cells, macrophages, monocytes, and neutrophils. Cell surface receptors, such as the B cell receptor (BCR) and FcγRIIa, facilitate the endocytosis of nucleic acid containing material or immune complexes (31, 32). Chromatin-containing immune complexes can stimulate B cells up to 100-fold more effectively than complexes without nucleic acids apparently due to collective engagement of BCR and TLR (31–34). Thus, dual engagement of the BCR and the TLR can induce abnormal activation of B cells and break immune tolerance. In human lupus, an increased proportion of B cells and monocytes expressed TLR-9 among patients with active SLE compared to patients with inactive disease (35). TLR activation in combination with T cell derived IL-21 markedly increased B cell differentiation into plasma cells (36).

**Figure 2:**
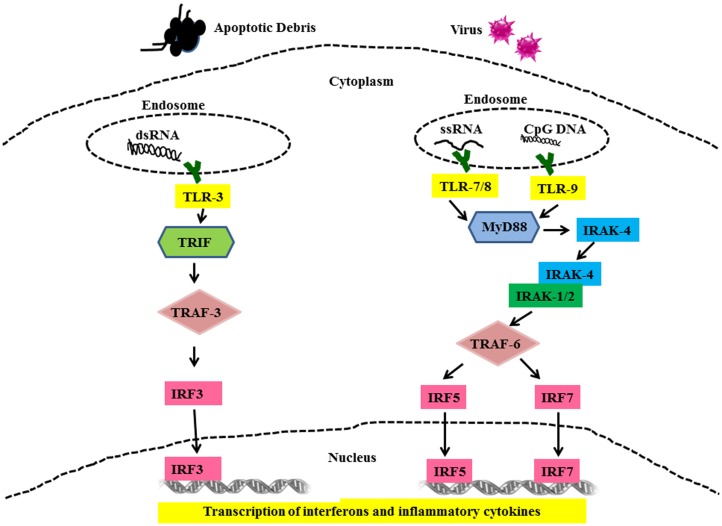
**Toll-like receptor mediated signaling in SLE**. Cells use TLRs as sensors to detect the presence of viruses and apoptotic debris via TLR-3, -7, -8, and -9. Nuclear material is trafficked to the endosome triggering TLRs signaling. Binding of cognate ligands to these TLRs recruits MyD88, a main signaling intermediate involved in TLR-7, -8, and -9 signaling. MyD88 recruits interleukin-1 receptor-associated kinase (IRAK)-4. IRAK-4 binds and phosphorylates IRAK-1, which in turn recruits Tumor necrosis factor (TNF) receptor-associated factor (TRAF) 6. IRF5 and IRF7 are then shuttled to the nucleus and these events set the stage for the transcription of IFN-α and other pro-inflammatory cytokines. TLR-3 signaling is MyD88-independent and utilizes TRIF and TRAF3 as signaling intermediates finally leading to activation of IRF3 and production of IFN-α and other pro-inflammatory cytokines.

All TLR family members, including TLRs-7, -8, -9 are type I membrane proteins composed of a ligand-binding ectodomain containing 18–25 tandem copies of leucine-rich repeats (LRRs), a transmembrane domain, and a conserved cytoplasmic toll/interleukin-1 receptor (TIR) domain. Ligand-induced dimerization and conformational rearrangement of the TIR domains leads to the creation of two symmetry-related sites which allow binding of the cognate signaling adaptor molecules (37, 38). Two main adaptors are utilized by TLRs, namely Myeloid Differentiation Factor-88 (MyD88) (TLR-7, -8, and -9) and TIR domain-containing adaptor inducing IFN-β (TRIF) (TLR-3). These adaptors mediate the recruitment of a series of kinases that lead to the formation of specific macromolecular signaling platforms for inflammatory reactions. IL-1 receptor-associated kinase 4 (IRAK-4) is recruited to MyD88 and is activated after recruitment (38). IRAK-4, in turn, activates IL-1 receptor-associated kinase 1 (IRAK 1) via phosphorylation (39, 40). These activated kinases recruit tumor necrosis factor receptor-associated factor 6 (TRAF-6), which is an E3 ubiquitin ligase required for activation of NFκB by freeing it from its inhibitor, I kappa B (IκB) (41). In addition to this, interferon regulatory factors (IRFs) IRF5 and IRF7 are recruited to the MyD88/IRAK/TRAF6 complex, where they become phosphorylated and activated (42, 43). Ultimately, the transcription factors NFκB and IRF5 and IRF7 are activated and translocate into the nucleus where they initiate gene transcription and production of pro-inflammatory cytokines and type I IFN (Figure 2) (43–45). Unlike TLR-7, -8, and -9, TLR-3 signaling is MyD88-independent and utilizes adaptor protein TRIF (46). TRIF also recruits additional proteins necessary for downstream signaling, including TRAF-family member-associated NFκB-activator-binding kinase 1 (TBK1), TRAF3, and receptor-interacting protein 1 (RIP1) (40). TRIF interaction with TBK1 is necessary for the activation of IRF-3, which is a transcription factor involved in the production of interferon beta (IFN_β_). (47). TLR-3 can also activate NFκB by the interaction of TRIF with TRAF-6 or RIP1 (40, 48) leading to up-regulated IFNα production and secretion of other pro-inflammatory cytokines.

## Genetic Factors Associated with TLR-Dependent IFNα Pathway in Lupus

One of the most striking immune system abnormalities in SLE patients is the frequent up-regulation of the type I IFN pathway (49, 50). IFNα is critical player in SLE progression and severity, and has been shown to induce the production of autoantibodies when administered to non-SLE patients (12, 51). An interesting report describes remission of SLE in a patient which was attributed to unresponsiveness to both TLR-7 and -9 stimulation after development of common variable immunodeficiency – (CVID-) like disease (52). Genetic variations in many of the components of the TLR signaling pathway have been associated with SLE, such as TLR-7, IRF5, IRF7, IRF8, IRAK1, and TNFAIP3 (53–59). Three of the nine genes in the IRF family have been genetically associated with SLE (60). Additionally, some of these genetic polymorphisms have been associated with increased type I IFN in SLE patients, supporting the idea that these genetic variations modulate the output of the TLR pathway (42, 60–64). The implication of these genes in SLE strongly supports the primary relevance of the TLR and IFNα pathway in the disease phenotype (63, 65). Additionally, many of these genetic polymorphisms in the TLR pathway are associated with the formation of autoantibodies (62–64, 66), supporting the concept of a feed-forward loop in which genetic variations in the TLR pathway enhance autoantibody production, and then the autoantibodies form immune complexes which stimulate the TLR pathway and result in increased type I IFN production in the setting of the same genetic variations. The TLR pathways are important in B cell maturation, and it is possible that genetically programed TLR pathway over-activity could promote autoantibody formation in B cells. Then after immune complexes are formed, these stimulate the TLR pathway in DCs and macrophages, and the same polymorphisms promote increased cytokine output from these cells.

## Toll-Independent Signaling in Lupus

### Signaling through RIG-1 like receptors in lupus

After viruses enter the cytoplasm and start replicating, infected host cells can sense and activate anti-viral responses in response to viral nucleic acids. This sensing occurs in the cytoplasm, and is independent of the cell surface and endosomal TLRs. Thus far, three cytosolic RNA helicases have been identified, RIG-I (retinoic acid – inducible gene I), MDA5 (melanoma differentiation – associated gene 5), and LGP2 (laboratory of genetics and physiology 2) that act as RNA sensors to mediate TLR-independent IFN-α/β induction in the presence of replicating RNA viruses (37, 67). Unlike membrane-bound TLRs, RLRs reside in the cytoplasm and sense cytoplasmic RNA. RIG-I contains tandem caspase recruitment domain (CARD)-like regions at its N-terminus and the central DExD/H helicase domain which has an ATP-binding motif and a C-terminal repressor domain which binds to RNA (68, 69). MDA5 contains tandem CARD-like regions and a DExD/H helicase domain, but it is unknown whether the C-terminal region of MDA5 really functions as repressor domain. LGP2 contains a DExD/H helicase domain and a repressor domain, but lacks the CARD-like region. LGP2 was suggested to be a negative regulator of RNA virus-induced responses, because the LGP2 repressor domain binds to that of RIG-I and suppresses signaling by interfering with the self-association of RIG-I (70, 71). Findings suggest that RIG-I and MDA5 have specificities in their detection of RNA viruses, through recognition of distinct viral RNA structures. RIG-I can recognize ssRNA bearing a 5′-triphosphate moiety (72, 73). In the case of self-RNA, 5′-triphosphate structures are removed or masked by a cap structure, which suggests a discrimination mechanism between self- and non-self RNA. RIG-I and MDA5 can distinguish dsRNA by size; RIG-I can bind short dsRNA whereas MDA5 can bind long dsRNA (74). Although LGP2 was considered a negative regulator, LGP2-deficient mice exhibited complicated phenotypes (75) and higher levels of type I IFN in response to polyinosinic: polycytidylic acid (Poly I:C) and vesicular stomatitis virus (VSV), but decreased type I IFN following encephalomyocarditis virus (EMCV) infection, suggesting that LGP2 can negatively or positively regulate RIG-I and MDA5 responses depending on the type of RNA virus (75).

**Figure 3:**
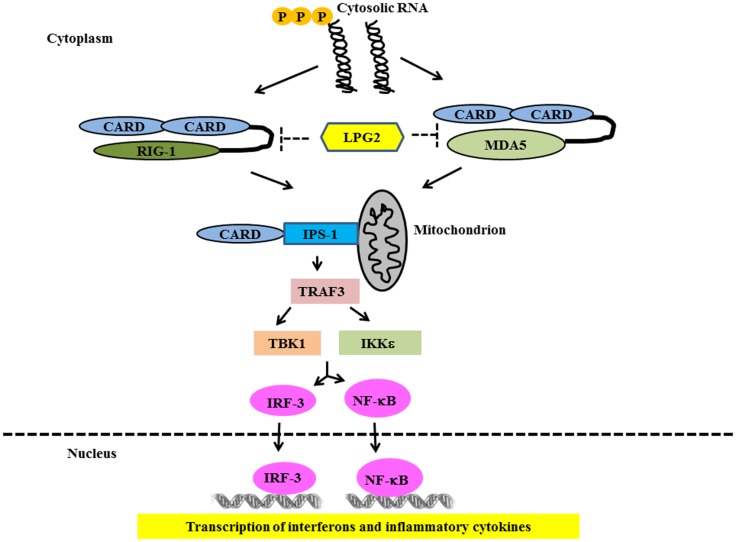
**Signaling through RIG-1 like Receptors in lupus**. Following recognition of the cytosolic RNA, RIG-I, and MDA5 associate with the adapter IPS-1 via CARD-like domains. IPS-1 is localized to the mitochondrion and initiates signaling leading to activation of IRF3 and NFκB that finally lead of over-production of type 1 IFN and other inflammatory cytokines.

Ligand binding to RLRs induces conformational changes leading to association with mitochondrial-associated IFN-β promoter stimulator 1 (IPS-1) through card-card domain interactions (76–79). IPS-1 then recruits TRAF3, which activates TANK-binding kinase 1 (TBK1) and IκB kinase (IKK) – related kinases IKKϵ (80). This leads to the phosphorylation and nuclear translocation of IRF-3 and -7 resulting in the transcription of IFN type 1 genes (81, 82) (Figure 3). IPS-1 also interacts with FAS-associated death domain protein (FADD) and receptor-interacting protein 1 (RIP-1) (76), which activate caspase-8 and caspase-10, resulting in NF-κB activation and production of inflammatory cytokines (83, 84). Genetic studies in SLE have strongly implicated the RLR pathways in SLE susceptibility. Variants in both MDA5 and IPS-1 have been associated with SLE susceptibility and with altered activation of the type I IFN pathway in SLE patients *in vivo* (85, 86). This again supports the idea that multiple nucleic acid recognition pathways are involved in SLE pathogenesis.

### Signaling through nucleotide binding and oligomerization domain (NLR) receptors in lupus

The NOD (NLR) family of receptors are key molecules that drive inflammatory responses by forming a multi-protein complex called “inflammasome.” The inflammasome drives the processing and release of cytokines such as the pro-inflammatory cytokines IL-1β and IL-18. Several inflammasome complexes have been identified in recent years. Of the known inflammasomes, NLRP3, absent in melanoma 2 (AIM2), and IFN inducible protein 16 (IFI16) inflammasomes have been linked to immune responses to intracellular DNA, as well as bacterial and viral infections (87). IL-1β is important in activating neutrophils, macrophages, DCs, and T cells, whereas IL-18 is crucial for IFN-γ production by NK cells and T cells (88). IL-1β and IL-18 are regulated at both transcriptional and post-translational levels. Upon transcriptional induction by TLRs and other sensor systems, IL-1β and IL-18 are synthesized as inactive precursor proteins, which are subsequently processed by the cysteine protease caspase-1 (IL-1β converting enzyme) (89). Conversion of procaspase-1 into an enzymatically active form, caspase-1, occurs upon formation of a multi-protein inflammasome complex (89). Previous reports have suggested that the NLRP3 inflammasome is involved in mediating the inflammatory responses to both DNA and RNA viruses (90, 91). In human SLE macrophages, NETs induce robust activation of the NLRP3 inflammasome (92).

**Figure 4:**
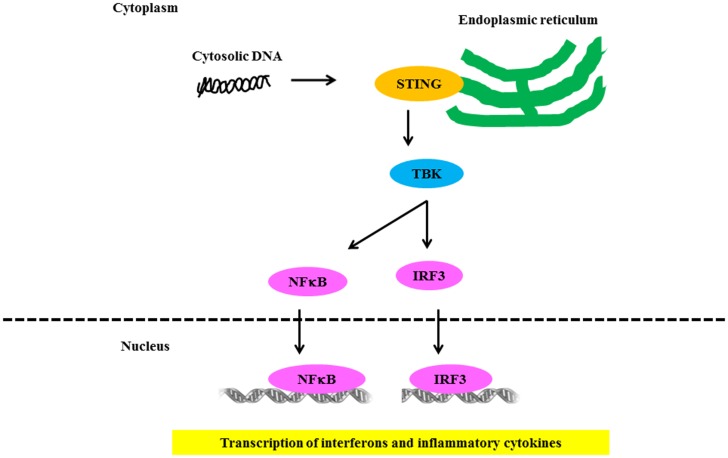
**Signaling through NLR receptors in lupus**. Intracellular DNA following microbial infection or phagocytosis of immune complexes can potentially trigger the assembly of NLRs. The nucleic acid-induced signaling pathway converges on the adaptor STING and the kinase TBK1, which phosphorylates IRF3 to mediate downstream signaling events leading to transcriptional induction of type 1 IFN and other inflammatory cytokines.

Several groups independently identified AIM2 as a receptor for cytosolic DNA that leads to caspase-1 activation and IL-1β secretion (93, 94). AIM2 binds cytosolic DNA of self and non-self origin, including bacterial, viral, and mammalian DNA, in a sequence-independent manner (95). Recent evidence indicates that the AIM2-related protein IFI16 also forms an inflammasome complex following Kaposi sarcoma – associated herpes virus infection of endothelial cells (96). Several groups independently identified STING as a key component of the DNA-sensing pathway (97, 98). STING/MITA translocates to perinuclear regions where it interacts with TBK1 to relay downstream signals to IRF3 (Figure 4). STING deficiency in macrophages or DCs leads to a markedly impaired type I IFN response to B-DNA and immunostimulatory DNA or to infection with DNA viruses, including HSV-1, human CMV, and vaccinia virus (97, 98). Initial studies showed that STING also interacted with components of the RNA-recognition machinery, such as RIG-I, where it was linked to type I IFN induction in response to VSV, a negative-strand RNA virus (97, 99). Murine models support the relevance of AIM2 in susceptibility to lupus-like disease in the NZB × NZW mouse (100).

## Other Cytosolic Nucleic Acid Sensors

### DNAse-I, II, and III

Production of type I IFN and inflammatory cytokines are important for protecting the host against infections; however overstimulation of innate immune pathways can induce autoimmune disease (101). Normally, host nucleic acid is limited to the nucleus and mitochondria whereas; host cellular DNA/RNA sensors are localized in the cytoplasmic compartment. Thus, accidental activation of inflammatory cytokine pathways by host defense sensors is largely averted. However, faulty clearance of self-nuclear material from apoptotic/necrotic bodies can cause improper activation of cytokines including type I IFN production.

One level of self-defense is provided by cellular endonucleases, such as Dnase-I, Dnase-II, and Dnase-III/Trex-1, which are involved in the clearance of extracellular, lysosomal, and cytosolic DNA, respectively. Genetic deficiencies of Dnase-I have been identified in SLE patients (102), and *Dnase I* – deficient mice develop a lupus-like syndrome (103). Dnase-I defects lead to the accumulation of extracellular DNA produced by apoptotic and necrotic cells, which is immunogenic and can lead to type I IFN production (101, 104). Dnase-II is expressed in lysosomes, where it degrades DNA from engulfed apoptotic/necrotic cells (105). Dnase-II knockout mice are embryonically lethal. However, they are viable on the IFNR1 knockout background, indicating that type I IFN mediates the lethality of Dnase-II genetic deficiency (101, 106). This finding supports the concept that inefficient nucleic acid degradation promotes type I IFN excess and subsequent SLE disease. Dnase-III is another nuclease that is normally involved in the clearance of cell-intrinsic ssDNA (107, 108). DNAse-III is 3′-5′ exonuclease and is localized to the endoplasmic reticulum. In the absence of DNAse-III, there is an accumulation ∼60-bp ssDNA, believed to be produced during replication, which leads to the activation of ATM-dependent DNA-damage associated checkpoint pathways (109). Stetson et al. (110) revealed a role for DNAse-III in preventing cell-intrinsic initiation of autoimmunity. Trex-1 substrates are ssDNA, which are either the by-products of replication and/or reverse transcribed from endogenous retroelements. Loss of function mutations in the human DNAse-III gene cause Aicardi–Goutieres Syndrome (AGS) (111, 112). Different rare DNAse-III mutations also cause monogenic chilblain lupus, and common genetic variations in DNAse-III have also been associated with risk of SLE, suggesting that a common mechanism may underlie these disorders (113–115).

### Other DNA and RNA sensors

DNA-dependent activator of IRFs (DAI) is another cytoplasmic DNA sensor capable of activating IRF-3 and NF-κB, resulting in type I IFN production. DAI interacts directly with dsDNA *in vitro* and this interaction in turn enhances DAI association with IRF-3. DAI-induced IRF-3 phosphorylation is dependent on TBK1 (47, 116). Recently, Zhang et al. (117) reported that DAI expression is predominantly increased in SLE patients as well as in activated lymphocyte-derived self-apoptotic DNA (ALD-DNA)-induced lupus mice. ALD-DNA could induce the dimerization/oligomerization of DAI and activate DAI signaling pathways via regulating calcium signaling, thus resulting in aberrant macrophage activation and lupus nephritis, implying the possible mechanisms for the recognition and regulation of ALD-DNA-induced pathological macrophage activation in the context of SLE disease (117).

Recently, Kondo et al. (118) identified MRE11 as a sensor for exogenous dsDNA, which is required for STING trafficking and type I IFN induction. The report reveals that MRE11 contributes to recognition of a broad spectrum of dsDNA and MRE11-mediated intracellular DNA recognition is to respond to damaged host cells, rather than defense against foreign pathogens (118). DDX41 is another DExD/H-box helicase that can interact with synthetic dsDNA through the DEAD domain *in vitro* and DDX41 is required for DNA-dependent induction of type I IFN in myeloid DCs through a pathway dependent on STING and TBK1 (119).

Found in the cytoplasm, RNA polymerase III is known to transcribe AT-rich DNA into dsRNA transcripts characterized by uncapped 5′-triphosphate moieties. This can act as a ligand for RIG-I. Subsequently, RIG-I signals via IPS-1 to induce the expression of type I IFN and other cytokines (72, 120). Ku80 is an abundant nuclear protein that is known to bind dsDNA with high affinity.

A recent study (121) identified Ku70, as the newest member of the cytosolic DNA-sensing machinery with in IFN production. Ku70 was identified as a DNA-binding protein in HEK-293 cells by DNA-affinity purification followed by mass spectrometry. Notably, Ku70 is involved in the production of type III IFN (λ_1_), but not type I IFN (α or β) in response to a variety of transfected DNA (>500 bp) in HEK-293 (121). It seems likely that we will continue to identify additional DNA and RNA sensors, and that some of these novel mediators will also play a role in SLE pathogenesis.

## Conclusion

In recent years, there has been tremendous progress in understanding how cells recognize and respond to microbial threats. Many DNA and RNA sensors have been identified that are dedicated to detection and elimination of microbial infection and clearing cellular damage. Sometimes these beneficial immune responses lose their fidelity and thus contribute to pathogenesis of autoimmune diseases. It is striking that many of the classical components of these pathways have been genetically associated with risk of SLE. This emphasizes the primary importance of nucleic acid handling and innate immune sensors in the pathogenesis of SLE. In SLE, it seems likely that stimulation of these pathways occurs via the combined contribution of microbial nucleic acids as well as self-tissue-derived stimuli. Work from our group and others supports a model in which immune complexes containing nucleic acid and free nucleic acid are a micro-environmental factor that cooperates with genetic variation in the nucleic acid sensing pathways to produce immune system dysregulation and risk of SLE (62). Understanding the molecular mechanisms of how the innate nucleic acid recognition system is dsyregulated in SLE will suggest new therapeutic avenues directed toward the inhibition of nucleic acid recognition by their sensors, downstream signaling events, and inhibition of end-stage mediators. This will lead to the new era of molecular medicine for the treatment of intractable autoimmune diseases like SLE.

## Conflict of Interest Statement

The authors declare that the research was conducted in the absence of any commercial or financial relationships that could be construed as a potential conflict of interest.
